# Mr. Smith's Reply to the Remarks of Mr. Tripe on the Statements in His Statistic Inquiry into the Frequency of Stone in the Bladder

**Published:** 1821-07

**Authors:** 


Mr. Smith's *reply to the remarks of* Mr. Tripe *on the statements in his Statistic Inquiry into the frequency of Stone in the Bladder.* [In a Letter to the Editor.]

IN my Statistical Inquiry into the Frequency of Stone in the Bladder, published in the eleventh volume of the Transactions of the London Medical and Chirurgical Society, I had in view no other object than a contribution to our general stock of knowledge, upon a subject confessedly in its infancy, and hitherto very much neglected.

In pursuance of my design, I endeavoured to conduct my plan, with as strict a regard to truth as possible; but it was to be expected that I was liable to fall into inaccuracies, since I could, in a great measure, submit to the public that only which I had received from others. The paper I considered and designated, a mere nucleus, around which, more correct information might be accumulated. As a member of the profession, therefore, so far from feeling hurt, I have to thank your correspondent, Mr. Tripe, of Plymouth, for having pointed out an error; and I hope very sincerely that his example will be followed by other surgeons in the United Kingdoms, and I pledge myself to profit by their remarks by an amended statement, when sufficient materials are elicited by their diligence. Long before I saw "the Remarks" upon the paper, it had been a cause of regret, that I had not given in the periodical Journals a notice of my intentions, and inserted a general invitation to the faculty to favour me with their experience. It was an unintentional oversight, which is, however, not altogether irreparable, if the several gentlemen will send to those useful publications memoranda of my errors, and of their own knowledge. As far as I am concerned, they cannot do me a more acceptable kindness.

That I may not, however, have to answer for more sins than I have committed, I beg to impress upon their minds, that my inquiry ends with the year 1817; also that my paper expressly alledges (page 45,) not only the general summary to be made up *according to the preceding reports*, but likewise presumes, *that several operations were performed which are not included in these records.*

In the conclusion, therefore, (page 50,) I have warned the reader that, to the cases on record, half as many again were to be added as a make-weight, for those cases which had not come to my knowledge. Thus, to the eighteen recorded cases for Devonshire, nine were added in the general computation for all England; making the numbers for that county, twenty-seven, in the eleven years which preceded January 1817. I would remark also, in justice to Mr. Barnes, that his statement to me ends in December 1816. Now Mr. Tripe's statement comes down nearly four years and a half later. In order, therefore, to be accurate, it will be needful for the latter gentleman to let us know the precise number he had cut to the end of the year 1816. I am sure, too, that Mr. Hammick will feel the necessity of not taxing his memory merely, but of giving us actual numbers, after due inquiry, up to that time. This will enable us to come at the absolute fact, and then we shall see the extent of the error, and be able to apply the correction.

That my paper is defective in information from two important stations upon the South Coast, does not lie entirely at my door. I addressed a circular "to the Surgeons of the Royal Naval Hospital at Plymouth," on or about the 14th of January, 1818, to which I received no reply. I am bound to consider that it did not reach its destination, feeling assured that had it fallen into the hands of Mr. Hammick, that gentleman's urbanity would have induced him to have complied with my request. The failure of my letter was the more unfortunate, as I should doubtlessly have been apprised of Mr. Tripe's operations, and those of the practitioners in the neighbourhood, which I should have considered a very valuable addition to the paper.

Being now placed as it were on my defence, I am obliged to add, however reluctantly, that at the same time I addressed one of the general circulars to Drs. Dodds and Vance, and the following is an extract from their reply.

"The surgeons of the Royal Hospital at Haslar, present their compliments to Mr. Smith, and beg leave to decline sending him the information he has desired in his letter, which they received a few days ago, because they cannot perceive that the acquisition of all the intelligence he looks for, can either be serviceable to the cause of humanity, or productive of any advantage to the surgical profession. If they were to afford matter for the composition of what they consider an useless publication, they would either be partakers of any disapprobation which the readers might be disposed to address, or be considered as tributary to an individual whose name they have never had the pleasure of hearing until now.

" *Royal Hospital, Haslar, Jan.* 24, 1818."

Upon the above I shall make only one comment, which is, that it forms the single refusal, and only exception to that polite, and even highly friendly, acquiescence on the part of those numerous gentlemen whom I took the liberty of troubling, and whose statements I am entitled to consider correct, unless where they shall be shewn, by satisfactory evidence, to be otherwise.*

38, *Park-street, Bristol; May* 20, 1821.

* We take the present occasion for stating that we shall be ready at all times to favour, as far as can be effected by means of this Journal, the acquisition of the information which Mr. Smith has solicited. It must be quite unnecessary to point out, in a particular manner, to medical men possessing common intelligence, the important benefits that may, with reason, be expected to result from the knowledge in question: but we may be permitted to suggest, that the utility of the Statistic accounts would be much increased by the addition of notices of peculiarities of diet and regimen of the people, more or less generally, of the districts to which they, respectively, relate. Information of this sort is an essential part of the Statistics which are calculated to contribute to our knowledge of the causes, and consequently favour our abilities for preventing, the disease. Every writer who has treated of stone in the bladder, in a comprehensive manner, has regretted the paucity of recorded information of this kind; and we think we should neglect our duty as the conductors of a Journal so long established as this, if we did not, now the matter is thus brought before us, join our solicitation to that of Mr. Smith, and at the same time express the gratification we feel on finding that the subject has engaged the attention of a medical man so well qualified by his generally acknowledged respectability and eminent professional talents, as well as by his reputation as an old and well experienced practitioner, to collect and to employ to advantage the information that is here required.—Edit.

